# Addressing Work-Related Issues in Medical Rehabilitation: Revision of an Online Information Tool for Healthcare Professionals

**DOI:** 10.1155/2016/7621690

**Published:** 2016-08-16

**Authors:** Matthias Lukasczik, Hans-Dieter Wolf, Christian Gerlich, Roland Küffner, Heiner Vogel, Silke Neuderth

**Affiliations:** ^1^Department of Medical Psychology, Medical Sociology and Rehabilitation Sciences, University of Würzburg, 97070 Würzburg, Germany; ^2^Faculty of Applied Social Sciences, Würzburg University of Applied Sciences, 97070 Würzburg, Germany

## Abstract

*Background.* Medical rehabilitation increasingly considers occupational issues as determinants of health and work ability. Information on work-related rehabilitation concepts should therefore be made available to healthcare professionals.* Objective.* To revise a website providing healthcare professionals in medical rehabilitation facilities with information on work-related concepts in terms of updating existing information and including new topics, based on recommendations from implementation research.* Method.* The modification process included a questionnaire survey of medical rehabilitation centers (*n* = 28); two workshops with experts from rehabilitation centers, health payers, and research institutions (*n* = 14); the selection of new topics and revision of existing text modules based on expert consensus; and an update of good practice descriptions of work-related measures.* Results.* Health payers' requirements, workplace descriptions, and practical implementation aids were added as new topics. The database of good practice examples was extended to 63 descriptions. Information on introductory concepts was rewritten and supplemented by current data. Diagnostic tools were updated by including additional assessments.* Conclusions.* Recommendations from implementation research such as assessing user needs and including expert knowledge may serve as a useful starting point for the dissemination of information on work-related medical rehabilitation into practice. Web-based information tools such as the website presented here can be quickly adapted to current evidence and changes in medicolegal regulations.

## 1. Introduction

Adverse working conditions and occupational stressors may impair work ability and complicate return to work after sickness or injury [1–6]. They may be associated with mental and somatic health problems, for instance, regarding stress-related somatic symptoms [7], depression [8], cardiovascular disease [9], or musculoskeletal disorders [10]. Addressing occupational issues in healthcare is therefore of great importance. Rehabilitation is a particularly suited and relevant setting: in many countries, its focus is on improving health status, treating chronic conditions, and/or altering their detrimental effects on activities and participation, in terms of the International Classification of Functioning, Disability, and Health (ICF) [11], which includes participation in working life.

Internationally, a growing number of vocational and/or medical rehabilitation concepts put a special emphasis on the interrelations of work-related variables, health, and work ability with return to work as a central rehabilitative outcome. These concepts vary in scope, target group(s), setting, or treatment elements (e.g., inpatient versus outpatient programs; workplace-related versus clinical interventions; coordination with actors involved such as healthcare providers and employers) [12–16]. For example, work-related programs have been introduced in inpatient medical rehabilitation centers in Germany that target patients with severe restrictions of work ability [17].

In view of current requirements specified by public healthcare payers (in Germany, e.g., by the statutory pension insurance as the main funding agency of work-related medical rehabilitation [18]), information on work-related rehabilitation concepts (including the available evidence) should be easily accessible to institutions and healthcare professionals working in this field, especially to those not (or less) familiar with this approach. The information provided should include resources and recommendations on how to implement vocationally oriented elements in medical rehabilitation.

Generally, information websites can be regarded as a widespread and useful educational strategy of dissemination [19]. Although less common than internet information tools that address patients [20], a web-based approach directed at healthcare professionals seems suitable to realize these elements. It is a low-threshold tool that is easy to adapt and can be accessed by a large number of users. A German study with general practitioners showed that physicians rate this format favorably [21]. However, online tools that support this implementation are largely lacking in rehabilitation, especially with respect to work-related programs. Currently, very few web-based information devices exist that offer information on rehabilitation topics (general information on rehabilitation [22]; preparation for rehabilitation; and follow-up/aftercare directed at patients [23, 24]).

Against this background, a website informing healthcare professionals in rehabilitation centers on work-related rehabilitation in the specific context of the German medical rehabilitation system had been developed between 2009 and 2010 [17, 25], with subsequent minor revisions following in 2011 and 2012. In this initial phase, standardized descriptions of major work-related treatment components had been developed in a consensus process using Delphi techniques with 50 experts from different professions working in work-related medical rehabilitation (medicine, psychology, social work, occupational therapy, physiotherapy, administration, and sports science). Descriptions of work-related rehabilitation programs and concepts already implemented in rehabilitation centers had been obtained to establish a database of good practice examples. These included the following descriptive features: indication(s); main content and treatment elements; target group(s) (including inclusion/exclusion criteria); therapeutic goals; therapeutic staff/professions involved; required equipment. General information on work-related medical rehabilitation and its translation into practice had also been made available to users.

This paper describes a major revision and update of the website (which can be accessed at http://www.medizinisch-berufliche-orientierung.de/; content currently available in German only) carried out between October 2013 and December 2014. Its purpose was (a) to enhance the practical value of the website to healthcare professionals by adding new topics and resources based on user needs and expert input, including practical implementation aids and resources and additional good practice examples and (b) to update the information provided on the website, referring to the current state of knowledge relevant to the setting. In this context, we referred to recommendations from implementation research: consideration of user needs; incorporation of expert knowledge; inclusion of good practice examples and practical implementation resources and recommendations; user-friendly presentation of the current state of knowledge [26]. The interprofessional character of work-related rehabilitation was also addressed [27].

## 2. Methods

To realize these objectives, the following steps were carried out.

To update the current state of knowledge presented on the website, a systematic literature search was conducted, comprising the period from 2011 to 2014. It focused on German publications referring to the specific healthcare setting and target group of the website. The websites and publications of relevant institutions in the German healthcare system (statutory pension insurance scheme, statutory accident insurance scheme, and vocational rehabilitation providers) as well as German scientific journals that regularly publish papers on medical rehabilitation were searched. Additionally, English-language studies by German researchers published in international journals and overviews of topics relevant to the context of work-related medical rehabilitation were reviewed, using the following databases: MEDLINE; PUBMED; Cochrane Database of Systematic Reviews; PSYCINFO. As search terms, the following headings (representing the main topics from the previous version of the website) were used: work-related medical rehabilitation (basics/development); screening for vocational problems; functional capacity evaluation instruments; self-rating instruments; motivation to deal with vocational issues; core interventions in work-related medical rehabilitation (work hardening; occupational training/therapy; patient education groups with vocational focus; social work counseling; cooperation with external institutions).

All rehabilitation centers already providing good practice examples on the website were contacted (*n* = 28 inpatient medical rehabilitation centers of various indications located throughout Germany). They were asked to indicate via a short questionnaire (6 items) whether they preferred (a) refined search criteria in the database including all good practice examples; (b) changes of the website structure or certain sections; (c) the addition of any new topics; (d) a regular newsletter; (e) a version for mobile devices; and (f) other issues of importance that could be specified in a free-text field as needed. The questionnaire had been developed specifically for this project. It was designed to assess to what extent several potential usage options and changes on the website would be of interest to (current and/or potential) users (see above). Its content and items were discussed and consented by the research team. Questionnaire data were analyzed using descriptive statistics.

Clinics were also asked to provide an updated description of their work-related measures in case there were any changes in their programs. In addition, an online form corresponding to the questionnaire was implemented on the website to give users the opportunity to propose modifications.

The results of the questionnaire survey were discussed at two expert workshops held in February and March 2014 with *n* = 14 representatives from rehabilitation centers, health payers, and research institutions. The following institutions/actors were included (number of participants in parentheses): German statutory pension insurance (1); German statutory accident insurance (1); vocational rehabilitation centers (1); medical rehabilitation centers (indications: orthopedics; psychosomatics; neurology; cardiology; metabolic diseases (9)); universities (rehabilitation research; sports sciences (2)). The following professions were represented: medicine; psychology; sports science; social work; occupational therapy; physical therapy; rehabilitation education/sciences.

The experts compiled proposals to revise the structure and contents of the website. Moreover, they participated in the revision of text modules on the website. The expert workshops followed focus group techniques with several predefined key topics guiding the workshops (revision/modification needs; workplace descriptions; website structure/outline).

Based on the literature search, the results from the questionnaire survey of rehabilitation centers and the expert workshops, several new topics to be included in the modified website were identified (see Section 3). As a basis for the preparation of corresponding new text modules, a complementary national literature search was conducted. Publications referring to these topics within the context of the German healthcare setting were reviewed that were published between 2011 and 2014.

To ensure comprehensibility, accuracy, and topicality of the information presented, all existing text modules were also reviewed. During this process, several parts of the website were extensively restructured, rewritten, and updated (based on the literature search described above).

In order to extend the existing database of good practice examples (which included *n* = 52 descriptions on the “old” website; see above), *n* = 12 medical rehabilitation facilities from different indications were contacted and asked to make their work-related treatment concepts available as additional good practice examples, using a form to describe the respective measure.

## 3. Results

In the questionnaire survey, 19 of 28 rehabilitation centers (67.9%) indicated they preferred one or several changes on the website (Table 1).

Results were discussed during the expert workshops. Here, it was eventually decided not to launch a newsletter (as suggested by the majority of institutions) in favor of a regularly updated overview of training and further education activities in areas/specialties relevant to work-related medical rehabilitation. Also, the suggestion of a version for mobile devices was not pursued due to limited technological resources.

The following additions to the website in terms of new topics were consented in the expert workshops: information on requirements of health payers and social security schemes regarding work-related medical rehabilitation; information on workplace/job descriptions; glossary; practical implementation aids; and resources (including material available for download provided by rehabilitation facilities, e.g., team briefing checklists). The latter comprise information on how to prepare patients for rehabilitation (e.g., importance of patient motivation, screening for vocational problems), the actual implementation of work-related rehabilitation programs (e.g., qualification of therapeutic staff), and potential risks and pitfalls.

Since the information provided on the website addresses clinicians and therapists, a separate brief section was added that informs patients on the objectives of the website. In this section, a link to another website that specifically targets rehabilitation patients was supplemented. This website (which had been developed in an unrelated research project [24]) informs patients (i.e., laypersons) on all aspects of medical rehabilitation (including work-related programs) in plain words.

The section on diagnostic tools used in work-related medical rehabilitation assessment was updated and extended. It comprises short descriptions of 28 standardized instruments available in German with links providing further information and resources and download options for those instruments that are license-free (Table 2).

Other (minor) modifications and restructuring made during the revision are listed in Table 3, which summarizes the structure and sections of the modified website.

**Figure 1:**
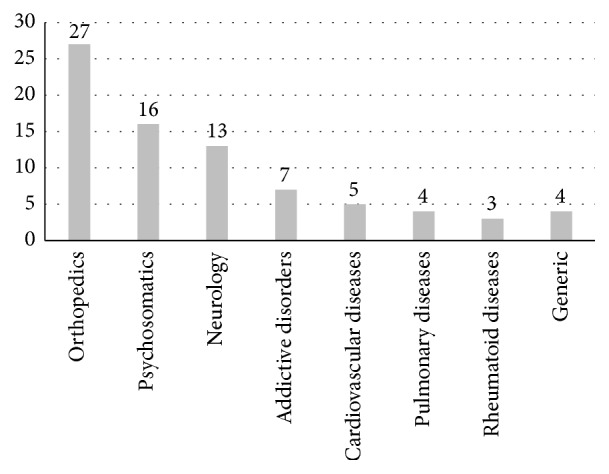
Indications represented in the database of good practice examples. Note: given is the number of examples per indication. The number of examples does not equal *n* = 63 as some examples relate to more than one indication.

Feedback regarding the modification of existing good practice examples was obtained for 50 descriptions. Clinics indicated modification needs in 29 of these examples, which were revised accordingly. The database including good practice examples was extended to 63 examples from seven indications (plus generic examples) including 11 new descriptions obtained from rehabilitation facilities throughout the revision process (Figure 1).

As mentioned above, all examples comprise detailed descriptions of the respective measure (e.g., target group; therapeutic goals; therapeutic professions involved).

The revised website was launched in January 2015.

## 4. Discussion

In this paper, we described the process of revising a website serving as an information tool for healthcare professionals in work-related medical rehabilitation (in Germany). The steps in this process followed recommendations for disseminating knowledge into practice, such as user involvement and the incorporation of expert knowledge [26]. These elements were realized by means of a survey of rehabilitation centers and the inclusion of an expert panel involved in the modification of contents. In doing so, we were able to specify topics regarded as essential by rehabilitation experts for the updated website.

Available evidence to be implemented into healthcare practice may cover varying types and levels of information, which may range from high-level research evidence to (good) clinical practice [38]. For the specific field of work-related medical rehabilitation in Germany, there is a lack of efficacy or effectiveness trials that can be regarded as steps or phases in the translation of research into practice [39]. Therefore, the aim of this project was to design a practical guide for clinicians and therapists in rehabilitation facilities and to provide them with information and “proven practice” specific to the German healthcare system, rather than presenting a systematic review of (international) evidence regarding vocationally oriented rehabilitation programs.

Web-based tools such as the website presented here offer several advantages. Their content and structure can be quickly revised and extended and is thus less prone to become obsolete. The specific information provided can be adapted to the respective healthcare setting and legal context. Moreover, users can easily access them. They also correspond to user expectations and preferences since clinicians themselves favor web-based information on rehabilitation-related contents [21]. As noted above, however, these tools are still uncommon in rehabilitation, especially with respect to work-related programs and are rarely accompanied by evaluation research.

Several limitations should be pointed out. First, there has been no user evaluation of the website so far. The revision process did not include a systematic quantitative assessment of the website by rehabilitation facilities or healthcare professionals. It is, however, important to examine whether the website actually reaches its target group as intended and to what extent users rate it as useful and informative. Therefore, future research should document the website's actual benefit to the targeted user groups and its suitability to transfer relevant information into rehabilitation practice. This should also include a larger number (and wider range) of rehabilitation facilities to allow a more detailed assessment, given that the revision of the website included only institutions already involved by providing good practice examples or practical implementation aids.

Second, as mentioned above, the website cannot (and does not claim to) provide a systematic and critical review of the international evidence, given its more “informational” focus including the requirements of national health payers and social security schemes. This fact in conjunction with the lack of higher-level evidence in the field of work-related medical rehabilitation (at least in Germany) limits the informative value of the website.

With regard to the clinics providing good practice examples and implementation resources, the centers took part on a voluntary basis. It cannot be ruled out that there is a “positive selection” of facilities with extensive expertise and a wide range of work-related rehabilitation programs (as compared to centers providing only basic programs or not yet offering work-related programs). Given the aim of the website to provide healthcare professionals with practical and field-tested information on how to establish and run work-related programs, we would not regard this as a major drawback.

Several clinics that took part in the survey on modification needs did not specify any needs. This leaves open the question whether these facilities were content with the status quo of the website or if they would prefer modifications other than those given in the questionnaire. As the free-text field was not used by these clinics to propose other suggestions, we can assume that they actually did not recognize a need for modification.

Finally, the long-term practical relevance of web-based information media depends on whether strategies to create a sustainable platform for this tool can be established (e.g., regarding personnel, financial resources). Implementation research has made rather few statements regarding this aspect [26, 40]. The website presented here was developed and revised in the context of several related research projects. This raises the question to what extent the resources necessary for the continuation of the platform can be ensured. This issue might be less problematic in other contexts, if, for instance, the tool is developed and maintained by a private company, social security agency, or health payer organization.

Research on the dissemination of evidence to professionals in medical rehabilitative practice is still relatively scarce. The development of information tools such as the website presented here may serve as a useful step to further establish work-related concepts in medical rehabilitation. This, however, must be accompanied and sustained by research that evaluates the respective programs and their effectiveness as well as the usefulness of associated information tools.

## Figures and Tables

**Table 1:** Feedback from rehabilitation centers regarding potential website modifications.

Newsletter	12
Refined search functions	7
Modified structure	5
New topics	5
Version for mobile devices	4
Other	0

Note: multiple answers possible.

**Table 2:** Assessments/diagnostic tools illustrated on the website with examples.

Type of assessment	Number of assessments described on the website	Examples
Screenings to identify patients with severe work-related problems/limitations	3	Screening-Instrument to identify the need for work-related medical rehabilitation (SIMBO) [28]; Würzburger Screening [29]

Functional capacity evaluation instruments (including profile comparison procedures that evaluate and compare work-related demands and functional capacities)	5	Isernhagen Work Systems FCE [30, 31]

Self-rating instruments (including the assessment of limitations of activities and participation and person-related context factors in terms of the ICF)	19	Disabilities of the Arm, Shoulder, and Hand Questionnaire (DASH) [32, 33]; Work Ability Index (WAI) [34, 35]; Effort-Reward Imbalance Questionnaire (ERI) [36, 37]

Note: screenings given as examples are currently available in German only.

**Table 3:** Overview of website structure and content.

		Content revised and extended	New content/topic
Background	Introduction to work-related medical rehabilitation	✓	
	Health payers' requirements/conceptual frameworks		✓
	Cooperation with external institutions (e.g., company physicians; vocational training institutes; career development centers)	✓	
	Promoting motivation in work-related medical rehabilitation	✓	

Components	Diagnostic tools/assessments	✓	
	Information on workplace/job descriptions		✓
	Work-related treatment components (as specified by the German pension insurance's profile of requirements [18]; e.g., work hardening; patient education groups with vocational focus)	✓	

Implementation	Good practice examples (database)	✓	
	Practical implementation aids and recommendations		✓

Service	Terms and definitions (glossary)	✓	
	Links and literature (cited literature, recommendations)	✓	
	Information in English		✓
	Information for patients		✓
	Feedback section (i) Submit a good practice example (ii) Submit information on workplace descriptions (iii) Recommend training/further education (iv) General feedback	✓	
	Further education and training (calendar of events)	✓	
